# The predictive value of weight gain and waist circumference for gestational diabetes mellitus

**DOI:** 10.4274/tjod.galenos.2019.03266

**Published:** 2019-10-10

**Authors:** Taha Takmaz, Ethem Serdar Yalvaç, Pınar Özcan, Ulaş Çoban, Ayşe Filiz Gökmen Karasu, Mehmet Ünsal

**Affiliations:** 1 Bezmialem University Faculty of Medicine, Department of Obstetrics and Gynecology, İstanbul, Turkey; 2 Bozok University Faculty of Medicine, Department of Obstetrics and Gynecology, Yozgat, Turkey; 3 İstanbul Şişli Hamidiye Etfal Training and Research Hospital, Clinic of Obstetrics and Gynecology, İstanbul, Turkey; 4 Universitiy of Health Sciences, Elik Zübeyde Hanım Women’s Diseases Training and Research Hospital, Clinic of Obstetrics and Gynecology, Ankara, Turkey

**Keywords:** Gestational diabetes mellitus, waist circumference, body mass index, weight gain, pregnancy

## Abstract

**Objective:**

The first objective of this study was to investigate the relationship between gestational diabetes mellitus (GDM) and gestational weight gain (WG), waist circumference (WC), prepregnancy, and gestational body mass index (BMI). The second aim of our study was to assess the ability of WG, WC, prepregnancy, and gestational BMI with special reference to their cut-off points on predicting the risk of GDM in pregnant women in Turkey.

**Materials and Methods:**

A total of 261 women who underwent screening for GDM with the 75-g glucose tolerance test (GTT) between 24^th^ and 28^th^ gestational weeks were included. According to the 75-g oral GTT results, women were classified into two groups: the GDM group and non-GDM group. The data collected included age, parity, plasma glucose level for fasting, 1- and 2-h tests, WC, prepregnancy and gestational BMI, prepregnancy weight, WG during pregnancy, gestational age at birth, and birth weight.

**Results:**

WC at 20-24 weeks of gestation, prepregnancy BMI, and gestational BMI had a predictive capacity for GDM. According to our results, optimal cut-off points for the best predictive value of GDM were WC of 100 cm with a sensitivity of 84% and specificity of 70%, prepregnancy BMI of 25 kg/m^2^ with a sensitivity of 81.8% and specificity of 76%, and gestational BMI of 28.3 kg/m^2^ with a sensitivity of 75% and specificity of 77.4%.

**Conclusion:**

The measurement of prepregnancy BMI, gestational BMI, and WC may be useful in predicting the risk of GDM. Pregnant women with increased prepregnancy BMI, gestational BMI, and WC measurements may be susceptible to the development of GDM.

**PRECIS:** The objective of this study was to investigate the relationship between gestational diabetes mellitus and gestational weight gain, waist circumference, prepregnancy and gestational Body moss index in pregnant women in Turkey.

## Introduction

Gestational diabetes mellitus (GDM) complicates 2-10% of all pregnancies. It is defined as varying degrees of glucose intolerance first diagnosed during pregnancy^(1,2)^. The diagnosis and management of GDM is extremely important because of the strong relationship between GDM and increased maternal and neonatal risks^(3,4)^. However, maternal and fetal outcomes in pregnancies complicated by GDM are strongly related to metabolic control^(5,6,7)^. GDM is probably a combination of genetic predisposition, metabolic factors, environmental factors, and lifestyle such as dietary habits and physical activity. There are several predictive markers for GDM including maternal obesity, gestational weight gain (WG), waist circumference (WC), and prepregnancy and gestational body mass index (BMI)^(8,9)^. Obesity is responsible for the central role in the pathogenesis of DM, a metabolic syndrome. WC and BMI seem to be more strongly linked to obesity. Increased BMI, a measure of general obesity, is a well-established risk factor for GDM. Increased WC, a simple and valid index of abdominal obesity, is an independent predictor for diabetes^(3)^. Maternal obesity is considered as an important predictive and modifiable marker in the short term for both maternal and fetal complications, including miscarriages, GDM, pregnancy-induced hypertensive disorders, macrosomia, maternal and fetal mortality, and cesarean sections, and in long-term risk factors for obesity and the metabolic syndrome in the child^(10,11,12,13,14)^. Moreover, maternal obesity, a major public health problem, has recently become more prevalent in line with the increase in the global prevalence of obesity. Maternal lifestyle is important for the reduction of GDM risk and the improvement of the total well-being of pregnant women and adverse pregnancy-related outcomes because pregnant women complicated with GDM have a higher prepregnancy and gestational BMI, a higher gestational WG, and a higher WC in general^(15,16)^. Thus, it should be an intervention focus. Turkish women may genotypically differ from other races and have different diet and lifestyle habits, which make them more vulnerable to obesity and GDM. However, body fat distribution is influenced by ethnicity. The first objective of this study was to investigate the relationship between GDM and gestational WG, WC, and prepregnancy and gestational BMI. The second aim of our study was to assess the ability of gestational WG, WC, and prepregnancy and gestational BMI, with special reference to their cut-off points, to predict the risk of GDM in pregnant women in Turkey.

## Material and Methods

### Study design and study population

The prospective cohort study was conducted at Department of Obstetrics and Gynecology of Etlik Zubeyde Hanim Women’s Health Teaching and Research Hospital between March 2015 and June 2015. The study protocol was approved by the institutional local ethics committee and institutional education and planning committee. It was based on the analysis of the results of 261 pregnant women who attended our outpatient department. A total of 261 women who underwent screening for GDM with the 75-g glucose tolerance test (2-h GTT) between the 24^th^ and 28^th^ gestational weeks as recommended by International Association of Diabetes and Pregnancy Study Groups (IADPSG) were included^(17)^. An abnormal GTT was defined as a single abnormal value that established the diagnosis (abnormal values defined by IADPSG: fasting ≥92, 1 h ≥180, and 2 h ≥153 mg/dL). According to the 75-g oral GTT (OGTT) results, women were classified into two groups: the GDM group (abnormal response, confirmed disease) and the non-GDM group (normal response, disease free). The inclusion criteria consisted of age 18-41 years with a single pregnancy. Exclusion criteria were maternal diabetes mellitus diagnosed before pregnancy, multiple gestations, preterm or postterm pregnancies, congenital malformation, the use of hyperglycemic agents (corticosteroids and thyroid hormones), a history of systemic medical conditions, a history of GDM or macrosomia, and pregnancy-induced hypertensive disorders. The data collected included age, parity, plasma glucose level for fasting, 1- and 2-h tests, WC, prepregnancy and gestational BMI, prepregnancy weight, WG during pregnancy, gestational age at birth, and birth weight.

### Measurements

WC was measured in the standing position, at the end of a normal expiration by using an inelastic tape (0.5 cm x200 cm) placed at the midpoint between the lower margin of the last palpable rib and the top of the iliac crest at the time of screening of GDM^(18,19)^. Prepregnancy and gestational BMI [(weight/height^2^ (kg/m^2^)] was calculated using the World Health Organization criteria as the most useful epidemiologic measure of obesity. Women were allocated into low weight (BMI <18.5 kg/m^2^), normal weight (BMI=18.5-24.9 kg/m^2^), overweight (BMI=25-29.9 kg/m^2^) and obese (BMI ≥30 kg/m^2^)^(20)^. Prepregnancy BMI was estimated based on self-reported prepregnancy weight. When prepregnancy weight was unknown, the weight measurement taken at the first prenatal clinic visit was used. Gestational BMI was determined based on the weight measurement taken at enrollment at the time of screening of GDM. WG was defined as the weight at enrollment (gestational weight) minus prepregnancy weight. WG percentage (WG%) was calculated as (gestational weight- prepregnancy weight/gestational weight) x100^(10)^.

### Statistical Analysis

Statistical analyses were performed using the Statistical Package for the Social Sciences, version 22 software package. Data were reported as mean ± standard deviation or number and percentage. P≤0.05 was considered significant. Normaly distributed continuous variables were assessed using independent Samples t-tests. Non-normally distributed metric variables were analysed using the Mann-Whitney U test. Spearman’s correlation was used to evaluate the associations of GDM with the variables of interest (WC, prepegnancy and gestational, BMI, WG). A multivariate logistic regression model was used to calculate the odds ratios (ORs) and 95% confidence intervals (CIs) for the likelihood of the prediction of GDM for WC, and prepegnancy and gestational BMI. Receiver operating characteristic (ROC) curves were constructed to calculate the sensitivity and specificity for different measures of prepegnancy and gestational BMI and WC in predicting GDM.

## Results

**Figure 1:**
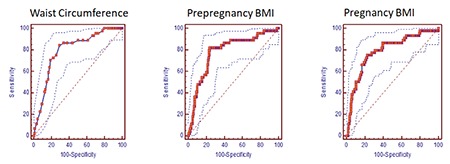
Receiver operating characteristic curve-sensitivity and specificity of waist circumference at 20-24 gestational weeks of pregnancy, prepregnancy and gestational body mass index to predict gestational diabetes mellitus BMI: Body mass index

Two hundred sixty-one women who underwent GDM screening with the 75-g OGTT were included in this study. The demographic and baseline obstetric characteristics of the women are shown in Table 1. There were 18 (6.9%) women with low weight (BMI <18.5 kg/m^2^), 155 (59.3%) women with normal weight (BMI=18.5-24.9 kg/m^2^), 52 (19.9%) women were overweight (BMI=25-29.9 kg/m^2^), and 36 (13.7%) women were obese (BMI ≥30 kg/m^2^). The mean ages of the women were 30.57±5.78 years in the GDM group and 26.34±5.58 years in the non-GDM group. Of the 261 women, 44 (16.85%) who had abnormal 75-g OGTT were allocated to the GDM group. There were statistically significant differences in age, WC, fasting plasma glucose concentrations, 1- and 2-h tests, prepregnancy and gestational BMI, prepregnancy weight, WG during pregnancy, and gestational age at birth (weeks) between the GDM group and the non-GDM group (Table 1) (p<0.01). In women with GDM, age, fasting plasma glucose concentration, 1- and 2-h tests, prepregnancy and gestational BMI, prepregnancy weight, WG during pregnancy, and WC were significantly higher, and gestational age at birth was significantly lower compared with women in the non-GDM group (Table 1. The GDM and non-GDM groups were similar with regard to birth weight. Multivariate logistic regression analysis revealed that there was a positive correlation between GDM and WC, and prepregnancy and gestational BMI. WC ≥100 cm [OR=8.36; 95% CI: (0.74-0.84); p<0.01], prepregnancy BMI ≥25 kg/m^2^ [OR=7.05; 95% CI: (0.72-0.82); p<0.01], and gestational BMI ≥28.3 kg/m^2^ [OR=7.2; 95% CI: (0.73-0.83); p<0.01] increased the incidence of GDM (Table 2). ROC curve analysis showed that prepregnancy BMI ≥25 kg/m^2^ predicted GDM with a sensitivity of 81.8% and specificity of 76% (AUC=0.78); gestational BMI ≥28.3 kg/m^2^ predicted GDM with sensitivity of 75% and specificity of 77.4% (AUC=0.78); and WC measurements ≥100 cm predicted GDM with a sensitivity of 84% and specificity of 70% (AUC=0.79) (Figure 1).

## Discussion

The results of our study demonstrated that the prevalence of GDM in our study population was 16.8%, and WC, prepregnancy, and gestational BMI, prepregnancy weight, and WG during pregnancy were significantly higher in women with GDM. Therefore, these markers may independently predict the risk of developing GDM. Moreover, there was a positive correlation between GDM and WC, and prepregnancy and gestational BMI; finally, our results may suggest new cut-off points for WC (≥100 cm), prepregnancy BMI (≥25 kg/m^2^) and gestational BMI (≥28.3 kg/m^2^) for the prediction of GDM.

GDM is undoubtedly associated with increased adverse maternal and neonatal outcomes^(21,22)^. The diagnosis and treatment of GDM absolutely results in decreased risks of maternal and neonatal adverse effects related to GDM^(23)^. Screening is important for the diagnosis of GDM because affected women are often asymptomatic. The screening of GDM may consist of either a one or a two-step approach. The IADPSG promoted the one-step approach (75 g, 2-h GTT at 24-28 weeks’ gestation) for the screening of GDM primarily based on the hyperglycemia and adverse pregnancy outcome trial data conducted to evaluate the association between mild hyperglycemia and adverse pregnancy outcomes in 2010^(24)^. The one-step approach was subsequently adopted by the American Diabetes Association in 2011^(25)^. We also adopted the one-step approach for the screening of GDM because of the cost-effectiveness and ease of application, and a relatively inexpensive future type 2 DM follow-up of the one-step approach. A retrospective study from Turkey demonstrated the prevalence of GDM as 4.8%, 8%, and 13.4% using the National Diabetes Data Group, Carpenter-Coustan and O’Sullivan two-step approach, respectively, and 22.3% with the IADPSG single-step approach. The study also reported that the prevalence of GDM increased with increasing age^(26)^. The pre-pregnancy or antenatally prediction of the risk of GDM with different strategies allows to reduce the incidence of GDM and improve maternal and infant health through the prevention of GDM (dietary, physical activity, behavior modification) or accurate diagnosis and appropriate treatment^(27,28)^. Overweight or obese, maternal age older than 35 years, excessive gestational WG, chronic hypertension, a history of GDM, strong family history of diabetes, polycystic ovarian syndrome, macrosomia, and stillbirth in a previous pregnancy, and high-risk racial/ethnic group are well-known risk factors for GDM^(29,30)^.

Maternal obesity is potentially a well-established risk factor for GDM and it is associated with some adverse maternal and neonatal outcomes such as preeclampsia, GDM, preterm birth, large-for-gestational-age babies or macrosomia^(31,32,33)^. Maternal obesity is principally defined based on the basis of WC and pre-pregnancy BMI. BMI is the most widely used method to determine total body fat and WC is a more practical measure for abdominal fat mass. A recent systematic review indicated that the risk of GDM was positively correlated with prepregnancy BMI, and the prevalence of GDM increased by 0.92% with every 1 kg/m^2^ increase in BMI [95% CI: (0.73-1.10)]^(8)^. A population-based study including 6795 women with GDM evaluated singleton pregnancies complicated by GDM in underweight and normal weight women. The authors reported 301 underweight women and 6494 women with normal BMI of 6795 women with GDM. According to their results, underweight women were younger, more often nulliparous, and had a lower incidence of birthweight >4000 g^(34)^.

However, several studies demonstrated that excessive gestational WG and gestational BMI might increase the risk of GDM^(35,36)^. Studies that assess cut-off points for prepregnancy and gestational BMI and WC based on race/ethnicity to predict GDM really important because body fat distribution is influenced by race/ethnicity. Madhavan et al.^(37)^ clearly demonstrated that there was a strong correlation between maternal obesity and obstetric complications. This pilot study conducted on Asian and Indian patients found that WC of 85.5 cm and a BMI of 24.3 kg/m^2^ had the best predictive value for GDM^(37)^. A cross-sectional study that included 240 women from Brazil showed that prepregnancy BMI (OR=4.21), gestational BMI (OR=3.17), and WC at 20-24 weeks (OR=4.02) were associated with developing GDM. According to the results of this study, WC at 20-24 weeks’ gestation is an important risk factor for GDM, and the range of 86-88 cm of WC has the best predictive performance for GDM^(38)^. Our results suggested that WC at 20-24 weeks’ gestation, prepregnancy BMI, and gestational BMI had predictive capacity for GDM. According to our results, the optimal cut-off points for the best predictive value of GDM are WC of 100 cm with a sensitivity of 84% and specificity of 70.9%, prepregnancy BMI of 25 kg/m^2^ with a sensitivity of 81.8% and specificity of 76%, and gestational BMI of 28.3 kg/m^2^ with a sensitivity of 75% and specificity of 77.4%.

### Study Limitations

The main strength of our study was its population-based and prospective nature with adjustment for the predictive capacity of traditional GDM risk factors and the selection of best predictive value of the cut-off points of prepregnancy and gestational BMI and WC for our country. The limitations of our study was the relatively small sample size.

## Conclusion

Our results confirm that the measurement of prepregnancy BMI, gestational BMI, and WC may be useful in predicting the risk for GDM. Pregnant women with increased prepregnancy BMI, gestational BMI, and WC measurements may be susceptible to the development of GDM. The cut-off points of prepregnancy BMI ≥25 kg/m^2^, gestational BMI ≥28.3 kg/m^2^ for being generally overweight, and WC ≥100 cm for central obesity were associated with increased risks of GDM. Determining these threshold points for prepregnancy BMI, gestational BMI, and WC measurements may be helpful in defining risky pregnant women in early pregnancy. Further well-designed randomized controlled trials are required to evaluate the use of these simple indicators of obesity for predicting GDM in pregnant women before these values can be used in clinical practice.

## Figures and Tables

**Table 1:**
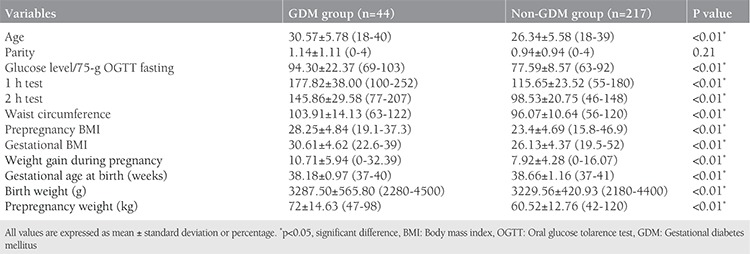
Demographic and baseline obstetric characteristics of groups

**Table 2:**
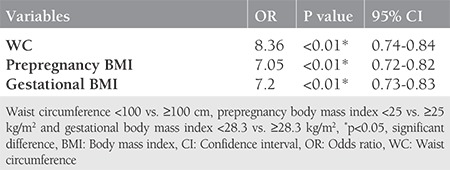
Multivariable analysis to predict to the presence of gestational diabetes mellitus
